# Antibiotic Treatment during Gestation Enhances Susceptibility to Mycobacterium tuberculosis in Offspring

**DOI:** 10.1128/spectrum.02491-22

**Published:** 2022-10-31

**Authors:** Donald D. Nyangahu, Courtney R. Plumlee, Bryan P. Brown, Colin Feng, Enock Havyarimana, Sara B. Cohen, Kevin B. Urdahl, Heather B. Jaspan

**Affiliations:** a Center for Global Infectious Disease Research, Seattle Children’s Research Institute, Seattle, Washington, USA; b Division of Immunology, Department of Pathology, Institute of Infectious Disease and Molecular Medicine, University of Cape Town, Cape Town, Republic of South Africa; c Department of Pediatrics, University of Washington, Seattle, Washington, USA; d Department Global Health, University of Washington, Seattle, Washington, USA; Geisel School of Medicine at Dartmouth

**Keywords:** BCG vaccine response, maternal microbiota, offspring microbiota

## Abstract

Whether antibiotic treatment during gestation impacts T cell immunity to vaccination in offspring is unexplored. Dams treated with polymyxin B (PMB) during gestation (Mg) displayed altered microbial communities prior to delivery compared to control dams (Mc). Differences in microbiota were also evident in pups born to polymyxin B-treated dams (Pg) compared to control pups (Pc). When pups were immunized with Bacille Calmette-Guerin (BCG), we observed no difference in TB10.4-specific T cells between Pc and Pg 4 weeks postimmunization. Significantly fewer splenic CD4 T cells from BCG-vaccinated Pg produced interleukin-2 (IL-2) upon stimulation, suggesting a possible functional deficiency. There was no difference in purified protein derivative (PPD)-specific IgG between Pc and Pg at this time point. However, when infected with Mycobacterium tuberculosis, Pg displayed significantly higher bacterial burden in the lung than Pc. Our results show that maternal PMB treatment during gestation may not impact splenic antigen-specific T cell responses following BCG vaccination but alters susceptibility to M. tuberculosis in offspring.

**IMPORTANCE** The composition of the pioneer microbiota that colonize the infant gut are determined by the mother. Polymyxin B-induced changes in the maternal microbiota during pregnancy impact the offspring gut microbiota but not vaccine-specific CD4 T cell response. However, when infected with Mycobacterium tuberculosis, offspring born to mothers with an altered gut microbiota are susceptible to infection compared to those born to mothers not exposed to antibiotics.

## INTRODUCTION

The early life microbiota has been shown to impact various aspects of infant development, including immune maturation (1–3) and metabolism (4, 5). The maternal gut microbiota has an important role in shaping intestinal colonization in offspring (6). We recently showed that altering the maternal microbiota during pregnancy impacts the offspring’s gut microbiota as well as immune development in a murine model (7). Furthermore, we and others have observed that changes in maternal gut bacterial composition during pregnancy or breastfeeding impacted total IgG and IgM levels in both mothers and offspring, suggesting that gut microbiota may impact antibody levels (8).

Recent data show that the microbiota is associated with altered vaccine responses (9). In human infants, an abundance of *Bifidobacterium* was positively associated with CD4 T cell proliferation to Bacille Calmette-Guerin (BCG) and tetanus toxoid vaccination at 15 weeks and 2 years of age (10). Considering that gut microbial composition in mothers during gestation influences developing infant immunity (7, 11), it is plausible that changes in the maternal microbiota during pregnancy may impact offspring responses to vaccination. BCG is a prototypic neonatal vaccine that is administered to infants predominantly at birth to protect against Mycobacterium tuberculosis. However, whether the maternal microbiota during gestation impacts offspring immunity to BCG is unknown. We have previously tested the impact of altering the Gram-positive bacterial community during pregnancy and lactation on offspring (7). Here, we sought to investigate how alterations in composition of Gram-negative bacteria during pregnancy impact the microbiota and immunity early in life. We hypothesized that antibiotic treatment during gestation impacts offsprings’ developing immunity and adaptive immune response. We tested this hypothesis by treating pregnant dams with antibiotics, which induced shifts in maternal gut microbial communities, and measuring offspring BCG T cell responses (TB10.4 specific). We used polymyxin B, which has activity against Gram-negative bacteria (12) and has poor oral bioavailability (13) and hence should have only direct effects in the maternal gut.

## RESULTS

### Impact of oral polymyxin B on maternal and offspring gut microbiota.

**FIG 1:**
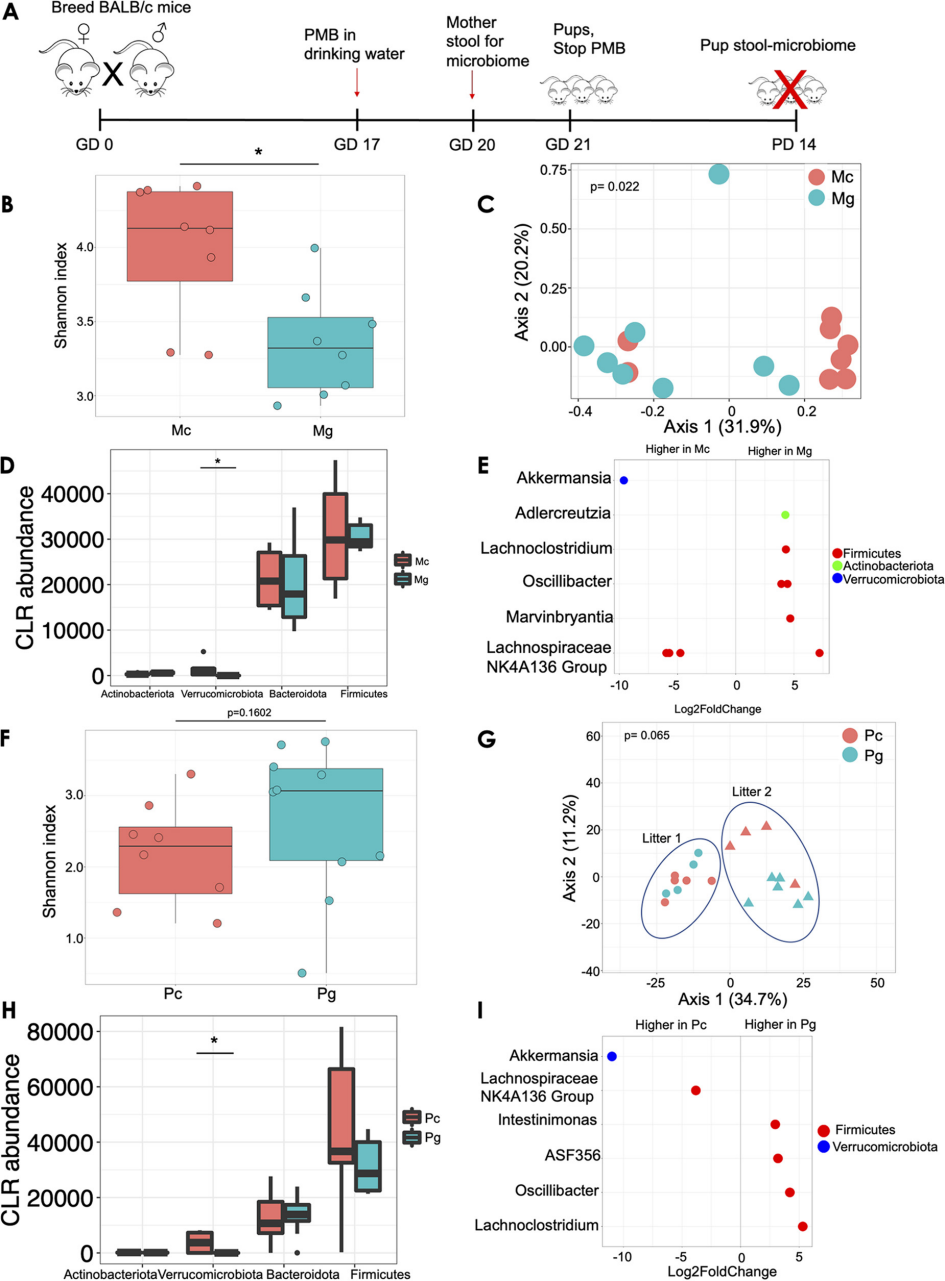
Oral antibiotics prior to delivery induce shifts in maternal and offspring gut communities. (A) Experimental design. Pregnant BALB/c dams were treated with polymyxin B in drinking water (1 mg/mL) starting at gestational day (GD) 17 until delivery, when antibiotic treatment was stopped. At gestational day 20, maternal stools were collected for 16S marker gene analysis. Pup gut microbiota was assessed at postnatal day (PD) 14. (B) Shannon alpha diversity between dams that received polymyxin B (Mg) versus control dams (Mc). (C) PCoA of maternal microbiota during pregnancy determined by the Bray-Curtis dissimilarity metric. (D) CLR abundance of bacteria in pregnant dams at the phylum level. (E) Differentially abundant genera by DeSEq2 during pregnancy. (F) Shannon alpha diversity of stool microbiota in pups born to polymyxin B-treated dams (Pg) and control pups (Pc). (G) PCoA of pup fecal microbiota determined by Bray-Curtis dissimilarity. (H) CLR abundance of pup microbiota at the phylum level. (I) Differentially abundant genera determined using DeSEq2 in pups. Pup microbiota were analyzed from two dams per group (two litters). Pregnant dams were separated and housed in individual cages prior to delivery. Data are representative of two independent experiments. *N* = 8 per group for dams and 9 to 10 per group for pups. *, *P* < 0.05.

In order to investigate the effect of the maternal microbiota on offspring, we treated pregnant dams with polymyxin B (PMB) in drinking water from gestation day (GD) 17 until delivery (Fig. 1A). PMB has narrow antibacterial spectrum activity against Gram-negative bacteria and has poor oral bioavailability (13, 14). We first confirmed that short-term oral antibiotics induced shifts in maternal gut microbial communities in dams during pregnancy by collecting stool samples from treated or untreated dams at GD 20 and analyzing the gut microbiota. Pregnant dams treated with PMB during gestation (Mg) had significantly lower stool Shannon alpha diversity (within-sample diversity) than untreated pregnant controls (Mc) (Shannon index, 3.19 versus 3.79; *P* = 0.011; Fig. 1B). We observed distinct clustering of Mc and Mg by grouping by principal-coordinate analysis (PCoA; between-sample diversity) of Bray-Curtis dissimilarity (permutational multivariate analysis of variance [PERMANOVA] *P* = 0.022; Fig. 1C). At the phylum level, we observed significantly reduced centered log ratio (CLR) transformed abundance of *Verrucomicrobiota* in Mg compared to Mc dams (Wilcoxon *P* = 0.025; Fig. 1D). Analyses of differentially abundant taxa using DESeq2 revealed the genus *Akkermansia* to be less abundant in Mg than in Mc (adjusted [adj] *P* < 0.001; Fig. 1E). *Adlercreutzia*, *Lachnoclostridium*, and *Oscillibacter* were significantly more abundant in Mg than in Mc (Fig. 1E; adj *P* < 0.001). We next assessed gut microbial composition in pups at postnatal day (PD) 14 to assess the effects of maternal gestational antibiotics on the offspring gut microbiome. Pups born to PMB-treated dams (Pg) exhibited increased Shannon alpha diversity compared to control pups (Pc), but the increase was not statistically significant (Shannon index, 2.64 versus 1.98; *P* = 0.16; Fig. 1F). Despite an obvious litter effect, we observed some clustering by group in Pc versus Pg by PCoA of Bray-Curtis distances (PERMANOVA *P* = 0.065; Fig. 1G). Akin to dams, Pg displayed significantly reduced CLR transformed abundance of *Verrucomicrobiota* compared to Pc (Wilcoxon *P* = 0.003; Fig. 1H). The genera *ASF 356*, *Oscillibacter*, *Lachnoclostridium*, and *Intestinimonas* were significantly more abundant in Pg than in Pc after controlling for litter (adj *P* < 0.001; Fig. 1I). We assessed total bacterial load in a subset of dams and offspring using BactQuant as previously described (15). There were no differences in bacterial load between Mc and Mg (see Fig. S1A in the supplemental material) or between Pc and Pg (Fig. S1B). In summary, oral administration of PMB prior to delivery induces compositional shifts in bacterial communities in the maternal gut (during pregnancy) and, to a lesser degree, in offspring’s guts 2 weeks postpartum.

### Impact of gestational antibiotics on inherent immunity in dams and offspring.

**FIG 2:**
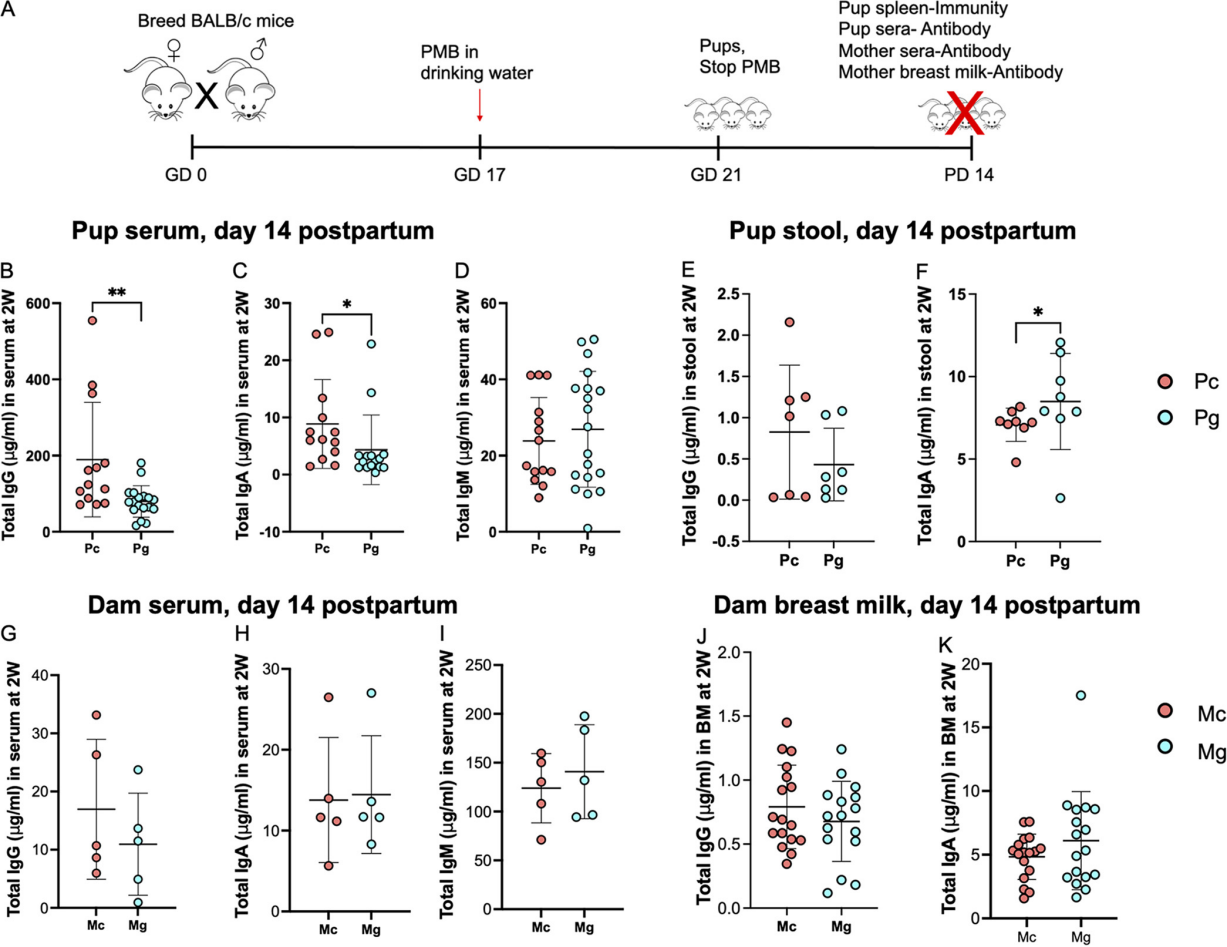
Alterations in the maternal and offspring gut microbiota impact total antibody levels. (A) Experiment schema. Pregnant BALB/c dams were treated with polymyxin B in drinking water (1 mg/mL) starting at gestational day (GD) 17 until delivery, when antibiotic treatment was stopped. At postnatal day (PD) 14, mice were sacrificed. Total antibody levels were measured in dam and pup sera and stool and in breast milk. Immune cells were profiled in pup spleens. (B to D) Total immunoglobulin concentrations in pup sera. (E and F) Antibody in pup stools. (G to I) Total immunoglobulin concentrations in dam sera. (J and K) Total immunoglobulin concentrations in breast milk. Shown as mean ± standard deviation (SD). Data are combined from at least two independent experiments. *n* = 4 to 5 per group for dams and 7 to 16 per group for pups. *, *P* < 0.05.

We and others have previously reported that alteration of the maternal gut microbiota during gestation alters postnatal inherent immune development in pups (7, 11). We assessed whether this was true with PMB treatment. We administered PMB to pregnant dams starting on GD 17 until delivery, as before, and measured growth and immunity outcomes in dams and offspring at PD 14 (Fig. 2A). There was no difference in body weight between Mc and Mg during gestation and lactation (Fig. S2A) or in their litter sizes (data not shown). Similarly, there were no differences in pup body weights at PD 14 (Fig. S2B). We observed no differences in splenic CD4 T cell frequency or number between Pc and Pg (Fig. S2C and D). Pg had significantly higher frequencies of splenic CD8 lymphocytes (23.5% versus 22.2%, *P* = 0.028) and follicular B cells (36.1% versus 34.1%, *P* = 0.016) compared to Pc pups, although the absolute numbers were not different (Fig. S2E to I). We observed significantly lower levels of total IgG (79.5 μg/mL versus 189.1 μg/mL, *P* = 0.003) and IgA (4.3 μg/mL versus 8.8 μg/mL, *P* = 0.010) in serum of Pg versus Pc (Fig. 2B to D). In pups’ stools, there was no difference in concentration of total IgG between Pc and Pg (Fig. 2E), but we observed significantly higher concentrations of total stool IgA in Pg versus Pc (8.4 μg/mL versus 7.2 μg/mL, *P* = 0.049; Fig. 2F), suggesting preferential retention of IgA in Pg guts. Since *de novo* antibody production in pups does not occur until after postnatal day 16 (16, 17), these observed immunoglobulins are likely of maternal origin. To assess whether differences in offspring antibody concentration were due to reduced maternal antibody, we measured total antibody concentrations in maternal serum and observed no difference in concentrations of IgG, IgA, and IgM between Mg and Mc (Fig. 2G to I). Similarly, there was no difference in IgG and IgA concentrations in breast milk (Fig. 2J and K), suggesting that factors other than maternal antibody levels contribute to reduced immunoglobulin concentrations in offspring. Incidentally, we also noted IgG concentrations to be several-fold higher in pups’ sera than that of dams’. To establish whether changes in offspring gut microbiota led to intestinal inflammation, we measured lipocalin-2 (LCN2), a marker of intestinal and metabolic inflammation and infection (18), and diamine oxidase (DAO), a marker of intestinal mucosal injury (19, 20), in pup serum. We observed no difference in concentrations of both LCN2 and DAO in sera of pups (Fig. S3), suggesting that local inflammation was not contributing to the differences in pup immunoglobulin. Overall, we found that PMB treatment during gestation had modest effects on offspring’s immunoglobulin levels.

### Effect of maternal gestational antibiotics on BCG vaccine response.

**FIG 3:**
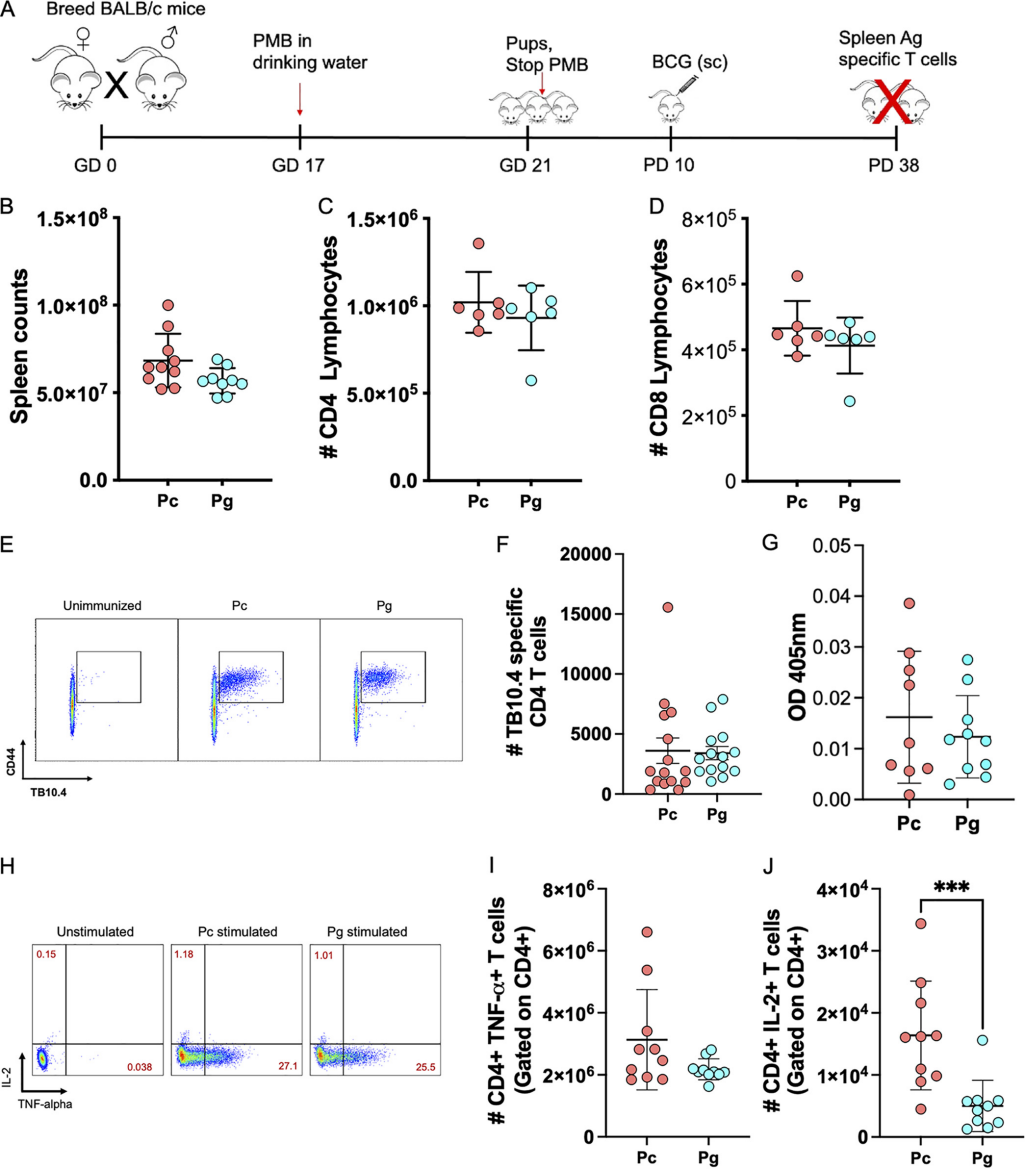
Maternal PMB does not impact the TB10.4-specific CD4 T cell response in offspring. (A) Experimental schema. Pregnant BALB/c dams were treated with polymyxin B in drinking water (1 mg/mL) starting at gestational day (GD) 17 until delivery, when antibiotic treatment was stopped. Pups were immunized with 10^6^ CFU of BCG subcutaneously at postnatal day 10, and antigen specific immunity was assessed 4 weeks postvaccination. (B) Splenic cell counts in BCG-immunized mice. (C and D) Absolute counts of CD4 and CD8 T cells in the spleen. (E) Representative flow plot of TB10.4-specific CD4 T cells. (F) TB10.4-specific CD4 T cells in spleens. (G) PPD-specific IgG in pup sera. (H) Representative flow plot of CD4 T cells producing TNF-α or IL-2. (I and J) Numbers of TNF-α- or IL-2-positive CD4 T cells. Shown as mean ± SD. Data show two or three independent experiments. *n* = 9 to 15 per group. *, *P* < 0.05.

The gut microbiota has been implicated in humoral vaccine responses in murine models (21) and associated with T cell responses in humans (10, 22). We next asked whether maternal antibiotic-related gastrointestinal tract (GIT) bacterial perturbations in Mg would impact offspring’s T cell response to vaccination. BALB/c Pc and Pg pups were immunized with BCG subcutaneously at PD 10, and BCG-specific immunity was measured 4 weeks postvaccination (Fig. 3A). There were no differences in total splenic cellularity or number of total CD4 T or CD8 T cells between BCG-immunized Pc and Pg (Fig. 3B to D). Moreover, we observed no significant difference in number of TB10.4-specific CD4 T cells between Pc and Pg (Fig. 3E and F). There was no difference in purified protein derivative (PPD)-specific IgG concentrations in serum between BCG immunized Pc versus Pg (Fig. 3G). Previous studies have provided extensive evidence that immunization of mice with BCG induced dominant Th1-type immune responses characterized by elevated expression of gamma interferon (IFN-γ), interleukin-2 (IL-2), and tumor necrosis factor alpha (TNF-α) (23–25). TNF-α is also known to be an important cytokine necessary for M. tuberculosis killing (26, 27). Therefore, to assess differences in functionality of the T cells in Pg versus Pc, we stimulated splenocytes for 6 h with PMA and ionomycin and stained for TNF-α and IL-2 intracellularly. We observed no difference in number of CD4 T cells producing TNF-α between Pc and Pg. In contrast, the number of CD4 T cells producing IL-2 were significantly decreased in Pg compared to Pc (*P* = 0.0007; Fig. 3J). Our data suggest that PMB treatment during gestation has no impact on total T cells or TB10.4-specific CD4 T cells but leads to reduction in IL-2 production in offspring splenic CD4 T cells 4 weeks following BCG vaccination.

### Altered offspring gut microbiota and M. tuberculosis control.

**FIG 4:**
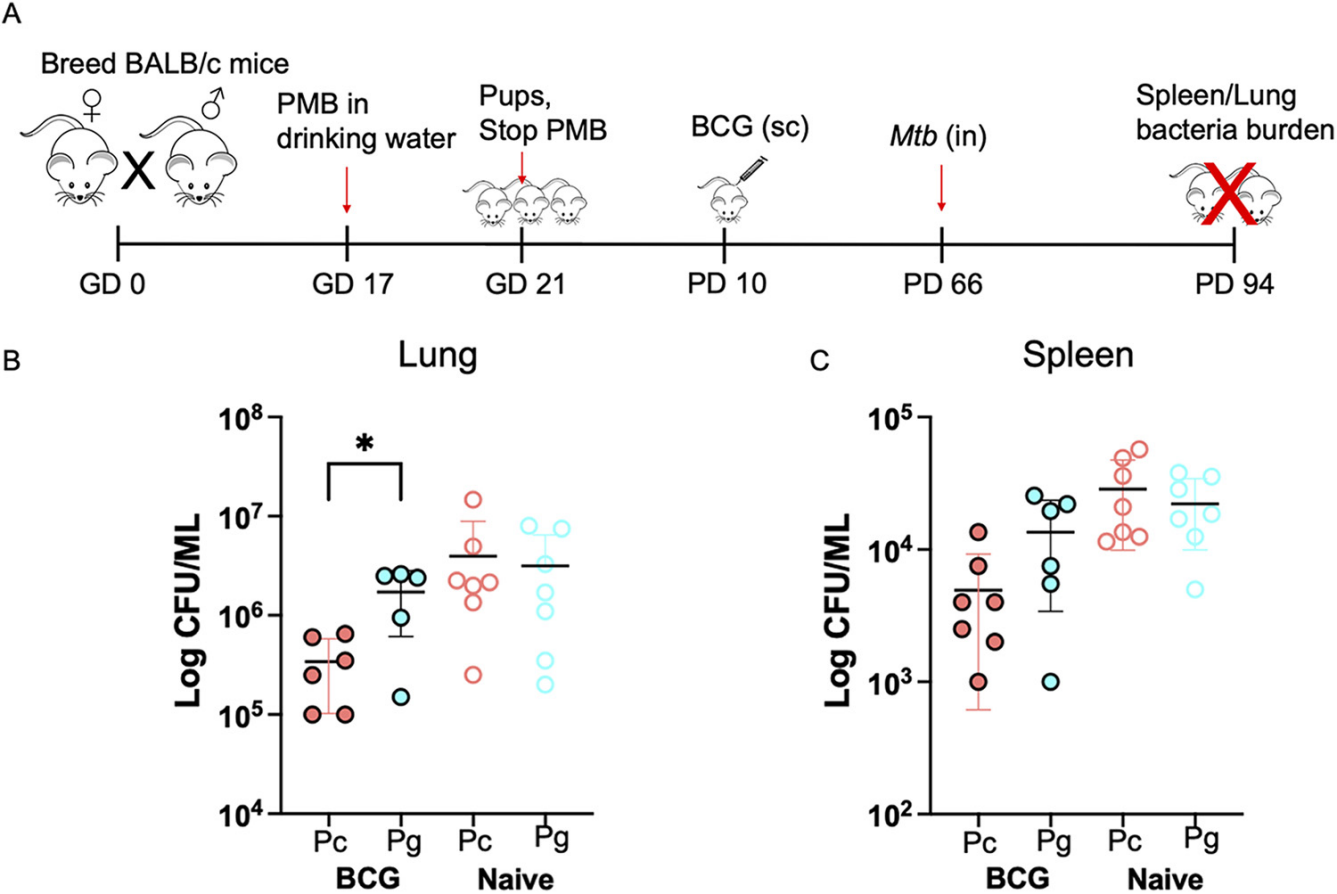
Progeny born to antibiotic-treated dams are susceptible to Mycobacterium tuberculosis infection. (A) Schema. Pups born to antibiotic breeders (Pg) or controls (Pc) were immunized with BCG at postnatal day 10 and challenged with M. tuberculosis 8 weeks postvaccination. Bacterial CFU were analyzed in lungs and spleens 4 weeks postinfection. (B) Bacterial burden in the lungs. (C) Bacterial burden in the spleen. Shown as mean ± SD. Data are representative of two independent experiments. *n* = 5 to 7 per group. *, *P* < 0.05.

Finally, we assessed whether the PMB treatment in dams impacted immunity to pathogen challenge in offspring. We infected pups with M. tuberculosis 8 weeks after BCG immunization (BCG administered at PD 10 in pups). Then, 4 weeks after challenge, we determined the bacterial CFU burden in the lungs and spleens (Fig. 4A). Interestingly, Pg displayed a 0.6 log higher lung CFU than Pc (*P* = 0.04; Fig. 4B). In addition, we observed higher bacterial CFU of disseminated bacteria in spleens of Pg versus Pc, but the difference was not statistically significant (Fig. 4C). Together, our results show that maternal oral PMB treatment during gestation increases susceptibility of offspring to M. tuberculosis challenge.

## DISCUSSION

Alterations in the maternal microbiota during pregnancy can be induced by antibiotics, diet, and maternal disease, among other factors (28–30), and these have consequences on offsprings’ developing microbiota. Here, we observed shifts in maternal gut bacterial communities as well as a reduction in microbial diversity following a short regimen of PMB during pregnancy. Maternal PMB led to significantly reduced abundance of *Verrucomicrobiota* (Gram-negative) in both dam and offspring guts, indicating that alterations in intestinal communities were consistent with the antibiotic’s spectral activity. However, despite this observation, pups born to Mg dams displayed a trend toward increased microbial diversity, which is opposite what we observed in dams, suggesting that PMB-induced effects of maternal gut communities are not necessarily transferrable. Total bacterial load was not different between Mg and Mc, consistent with previous work that showed no impact of PMB on stool microbiota density using a lower dose of PMB (31).

Microbiome seeding in early life coincides with the period in which vaccines are administered and the immune system is undergoing rapid development. Previous studies have reported a relationship between microbiota and vaccine responses (10, 21, 22, 32, 33). These studies examined the relationship of the microbiota on the host response to vaccination and not the transferred effects of the maternal microbiota during pregnancy. Whether the maternal microbiota during pregnancy determines infant T cellular vaccine responses is underexplored. Here, we observed no effect of an altered maternal gut microbiota on offspring BCG tetramer responses in the spleen 4 weeks following BCG vaccination. Our findings may be due to the limited effect of maternal PMB on offspring intestinal communities, and the outcome may be different in cases where antibiotics with a much broader spectrum are used to induce microbiological changes. Alternatively, we may not have captured changes in antigen-specific immunity due to the timing of our measurement (4 weeks) or the tissue analyzed (spleen only). Previous work has shown the mycobacterial-specific T cell response to vary in different tissues and with time (34). Previous studies have observed findings similar to ours with regard to antigen-specific antibody response. For example, Lynn et al. reported no differences in humoral responses to BCG in antibiotic-treated versus untreated infant mice (35). Of note, both dams and pups were treated with broad-spectrum antibiotics in that study, and BCG was administered 4 weeks postpartum. Here, we treated dams only with a Gram-negative targeting antibiotic for 5 days prior to delivery and administered BCG at PD 10 to mimic timing in human infants. Regardless, these data show that short-term perturbations of the maternal microbiota induced by PMB during pregnancy do not influence offspring antigen-specific CD4 T cells in the spleen 4 weeks following BCG vaccination.

Earlier studies reported that TB10.4-specific CD4 T cells correlate with M. tuberculosis protection (36). When we challenged pups with M. tuberculosis to test whether maternal antibiotics impacts M. tuberculosis immunity, Pg pups exhibited increased susceptibility to M. tuberculosis infection. This suggests that maternal PMB treatment may induce other immunological changes in the offspring that impaired M. tuberculosis immunity that we did not measure. While we have not shown a direct link between the maternal microbiota and offspring immunity, it is tempting to speculate that (i) inherited gut dysbiosis impacts BCG vaccine efficacy, which enhanced M. tuberculosis susceptibility, or (ii) PMB treatment of dams impacts the function of key immune cells in offspring required for effective M. tuberculosis clearance. Nadeem et al. showed that perturbations in the gut microbiota induced by antibiotics impaired BCG vaccine efficacy, leading to impaired generation of effector and memory T cells required for M. tuberculosis clearance (37). While we did not assess immunity in the infected Pc and Pg mice, it is plausible that maternal PMB treatment or altered offspring gut microbiota may have led to an impaired T cell response in M. tuberculosis-infected mice, hindering pathogen clearance. Indeed, we observed significantly reduced IL-2 production from splenic T cells in immunized Pg compared to Pc. It is plausible that this finding represents impaired functionality of the antigen-specific T cells to control M. tuberculosis, despite similar numbers of tetramer-positive cells. Furthermore, despite both Pc and Pg displaying similar numbers of TB10.4-specific CD4 T cells, control of M. tuberculosis did not reflect this, suggesting that splenic TB10.4-specific T cell responses measured 4 weeks postvaccination are not correlated with M. tuberculosis protection.

A limitation of our work is that we did not profile the kinetics of the antigen-specific T cells. Previous studies have shown that mycobacterial-specific T cells vary at different stages of infection (34, 38). A similar phenomenon is expected post-BCG vaccination. In this study, we assessed antigen-specific T cells only 4 weeks postimmunization. It is possible that this population could be different 8 weeks post-BCG immunization when we infected them with M. tuberculosis. Furthermore, it is likely that the kinetics of these antigen-specific T cells could be different in the lungs, a key site in M. tuberculosis control. Nonetheless, our data show that despite no differences in the number of antigen-specific CD4 T cells in the spleen, maternal PMB likely impairs other immunological factors, such as cytokine production, in Pg which enhance susceptibility to M. tuberculosis.

Taken together, we find that antibiotic-induced shifts in maternal intestinal communities do not impact the TB10.4-specific CD4 T cell response in the spleen of offspring but enhance susceptibility to M. tuberculosis infection. These findings represent a conceptual advance in our mechanistic understanding of how maternal gut microbiota may influence postnatal disease susceptibility in offspring.

## MATERIALS AND METHODS

### Mice.

All animal studies were approved by the Institutional Animal Care and Use Committee (IACUC) of Seattle Children’s Research Institute under animal protocol IACUC00328. Male and female BALB/c mice between 6 and 8 weeks old were purchased from Jackson Laboratories (Bar Harbor, ME) and maintained under specific-pathogen-free (SPF) conditions at Seattle Children’s Research Institute.

### Experiment 1: maternal and offspring gut microbiota.

Female BALB/c mice were bred at 7 weeks with BALB/c males. Males were removed from breeding cages after 8 days. Pregnant females were treated with polymyxin B (PMB; Cayman Chemical, MI) (1 mg/mL) in drinking water starting at gestational day (GD) 17 until delivery or left untreated. PMB was dissolved in autoclaved drinking water and refreshed after every 2 days. Dams treated with PMB are designated Mg, and the control dams are designated Mc. Pups born to Mg are designated Pg, while pups born to Mc are designated Pc. Fecal samples for microbiome analysis were collected at GD 20 from mothers and postnatal day (PD) 14 from pups.

### Experiment 2: maternal and offspring immunity.

Female BALB/c mice were bred and given PMB as described in experiment 1. At PD 14, all mice were sacrificed. Blood and stool (from both dams and offspring) and breast milk (pellets from pups’ stomachs) were collected to assess antibody levels by enzyme-linked immunosorbent assay (ELISA). Spleens were collected from pups for inherent immune analysis by flow cytometry.

### Experiment 3: BCG vaccination experiments.

Female BALB/c mice were bred and given PMB as described in experiment 1. Pups were immunized subcutaneously with BCG (10^6^ CFU/mouse) at PD 10. Immunized pups were sacrificed 4 weeks postvaccination to analyze BCG-specific responses. TB10.4 class II tetramers (I-A^d^ restricted) were used to measure antigen-specific CD4 T cells.

### Experiment 4: Mycobacterium tuberculosis infection experiments.

Female BALB/c mice were bred and given PMB as described in experiment 1 and immunized with BCG as described in experiment 2. BCG-immunized Pc and Pg mice were rested for 8 weeks prior to infection with Mycobacterium tuberculosis. Bacterial burden was analyzed in the lungs and spleens 4 weeks postinfection.

### BCG immunization.

BCG-Pasteur was grown in Middlebrook 7H9 broth supplemented with albumin dextrose catalase (ADC) enrichment, glycerol, and Tween 80 at 37°C to an optical density (OD) of 0.3 to 0.5. Bacteria were diluted in phosphate-buffered saline (PBS), and mice were injected with 100 μL subcutaneously (~10^6^ CFU) at PD 10. To analyze TB10.4-specific responses, mice were sacrificed 4 weeks post-BCG immunization.

### M. tuberculosis infections.

M. tuberculosis infection was performed via the respiratory route by using an aerosol infection chamber (Glas-Col) as previously described (39). Infections were performed with wild-type H37Rv M. tuberculosis and approximately 100 CFU delivered into the lung per animal. BALB/c BCG-immunized animals were infected 8 weeks postimmunization. Mice were euthanized 28 days after challenge. Lungs and spleens of individual mice were aseptically removed and homogenized separately in PBS with 0.05% Tween 80. Then, 10-fold serial dilutions were made and plated onto Middlebrook 7H10 agar plates. Colonies were counted after 21 days of incubation at 37°C to determine the CFU.

### Flow cytometry.

Single cell suspensions from spleen were prepared as previously described (7) from 14-day-old pups. Cells were then transferred to a V-bottom 96-well plate for immune staining. Splenocytes were stained for surface markers using lineage antibodies. T cells were stained with anti-CD3 Alexa 700 (BD, clone 500A2), anti-CD4 PerCPcy5.5 (BD, clone RM4-5), anti-CD8 BV510 (BD, clone 53-6.7), and anti-CD44 FITC (BD, clone IM7). B cells were stained with anti-CD19 PECy7 (BioLegend, clone 1D3), anti-B220 FITC (BioLegend, clone RA3-6B2), anti-CD21 APC (BD, clone 7G6), and anti-CD23 PE (BD, clone B3B4). Then, 50 μL of the antibody master mix cocktail (prepared in MACS buffer) was added per well, and cells were stained for 30 min at 4°C in the dark. Cells were then resuspended in acquisition buffer.

To measure BCG-specific responses, splenocytes were stained with phycoerythrin (PE)-conjugated major histocompatibility complex II (MHC-II) tetramer (I-A^d^) containing amino acids 73 to 88 of M. tuberculosis TB10.4 (NIH Tetramer Core Facility) for 1 h at room temperature. Cells were then labeled with anti-PE magnetic beads (Miltenyi Biotec) and enriched using magnetic columns (Miltenyi Biotec). Bound and unbound fractions were then stained with T cell markers at 4°C for 30 min. The dump channel on APCcy7 was used to exclude non-T cells (CD11c, CD11b, B220, F4/80). Cells were washed and resuspended for acquisition.

For intracellular staining of TNF-α and IL-2, 2 × 10^6^ cells were incubated with 500 ng/mL phorbol myristate acetate (PMA) and 1,000 ng/mL ionomycin for 6 h at 37°C. Brefeldin A (10 μg/mL) was added 2 h after incubation with PMA and ionomycin. Cells were first stained using fluorescently labeled antibodies against surface markers for 30 min on ice. Afterward, cells were fixed and permeabilized (eBiosciences buffer set) and stained with anticytokine antibodies (anti-IL-2 and anti-TNF-α; BD) for a further 30 min on ice. Cells were washed and resuspended in acquisition buffer. All cells were acquired on the LSRII instrument (BD) and analyzed with FlowJo (Tree Star, Ashland, OR).

### 16S rRNA gene sequencing and analyses.

DNA was extracted from stool as previously described (7, 40). Extracted DNA was used for library preparation. Briefly, the hypervariable region V3-V4 was amplified using modified universal primers, (Table S1) (41). Amplicon libraries were purified using AMPure XP beads (Beckman Coulter). Dual-index barcodes and Illumina sequencing adapters were added via sequential PCR using the KAPA HIFI kit (Roche), and amplicons were pooled in equal mass amounts. The library was quantitated via quantitative PCR (qPCR) using the NEBNext library quant kit for Illumina (New England Biolabs), and paired-end sequencing was performed on an Illumina MiSeq platform (2 × 300 bp).

For 16S rRNA gene data analyses, primers flanking the 16S rRNA amplicon reads were removed with Cutadapt using default parameters (42). Reads were then imported into R and processed using the DADA2 (v1.12.1) pipeline (43). Briefly, the pipeline performs quality checks and quality filtering, learns error rates, merges paired-end reads, constructs an amplicon sequence variant (ASV) table, removes chimeric sequences, and assigns taxonomy to ASVs using the SILVA (v138 release) reference database (44). Downstream analyses were performed in R using phyloseq (45), DESeq2 (46) and phylosmith (47).

### BactQuant to assess bacterial load.

The total bacterial load per stool was determined using a qPCR of the 16S rRNA gene (15). The BactQuant assay targets the V3-V4 region and gives an estimate of the total 16S rRNA copies per sample.

### Antibody ELISAs.

Sera and mucosal antibodies in dams and pups were measured using a mouse Ig ELISA kits (STEMCELL Technologies). Breast milk bolus was collected from the stomach of 2-week-old pups (48–50). Milk samples were resuspended at 100 mg/mL in PBS and spun at 10,000 × *g* for 10 min. Supernatants were carefully collected and used for ELISA. For fecal concentrations of antibody, fecal pellets were weighed and resuspended at 100 mg/mL concentration in PBS (8). Samples were spun at 16,000 × *g* at 4°C for 10 min, and were supernatants collected. Serum samples were diluted 1:30,000, while stool samples were diluted 1:10 prior to ELISAs. Absolute antibody levels in sera and mucosal sites were then determined by ELISA using the STEMCELL mouse ELISA kits per the manufacturer’s instructions. For determination of absolute antibody concentrations, standards were included in the assay (0.1 to 100 ng/mL), and sample OD values were fitted onto the standard curve.

For PPD-specific ELISAs, plates were coated with 100 μL of 1 μg/mL PPD overnight. Wells were blocked by adding 200 μL of 2% bovine serum albumin (BSA) in PBS for 2 h at room temperature. Serum samples from BCG-immunized animals diluted at 1:200 or 1:600 were added to the plate after washing and incubated at room temperature for 2 h. Plates were washed, and 100 μL of alkaline phosphatase-conjugated secondary antibody for IgG was added for an hour at room temperature. ELISA plates were washed and developed using *p*-nitrophenylphosphate substrate. Results are shown from a 1:200, dilution which gave a positive result after subtracting OD values for control serum (unimmunized animals).

### Other ELISAs.

Lipocalin-2 (R&D systems) and diamine oxidase (Cusabio) ELISAs on mouse serum were performed according to the manufacturer’s instructions.

### Statistical analyses.

Analyses were performed using Prism (GraphPad Software). A one-way analysis of variance (ANOVA) with Sidak’s multiple-comparison test or the Mann-Whitney U-test was used to analyze nonparametric data. For microbiome data, intergroup distance was assessed for significance using permutational multivariate analyses of variance (PERMANOVA) and homogeneity of group dispersion (51), and differences in alpha diversity were tested by Welch’s *t* test. In DESeq2 analyses to test for differential abundance, the alpha threshold was set at 0.05. Output *P* values were adjusted for multiple comparisons by Benjamini-Hochberg correction (46), and taxa with adjusted *P* values of less than 0.05 were considered to be significantly differentially abundant.

### Data availability.

16S rRNA sequences have been deposited at the SRA (accession number PRJNA785004) and are publicly available.
