# Supplementary material for: Antibiotic Treatment during Gestation Enhances Susceptibility to Mycobacterium tuberculosis in Offspring
Source: Microbiol Spectr. 2022 Oct 31;10(6):e02491-22. doi: 10.1128/spectrum.02491-22 (PMC9769670; doi:10.1128/spectrum.02491-22)
Supplement: Supplemental file 1 — Fig. S1-S3. Download spectrum.02491-22-s0001.pdf, PDF file, 0.6 MB [file spectrum.02491-22-s0001.pdf]

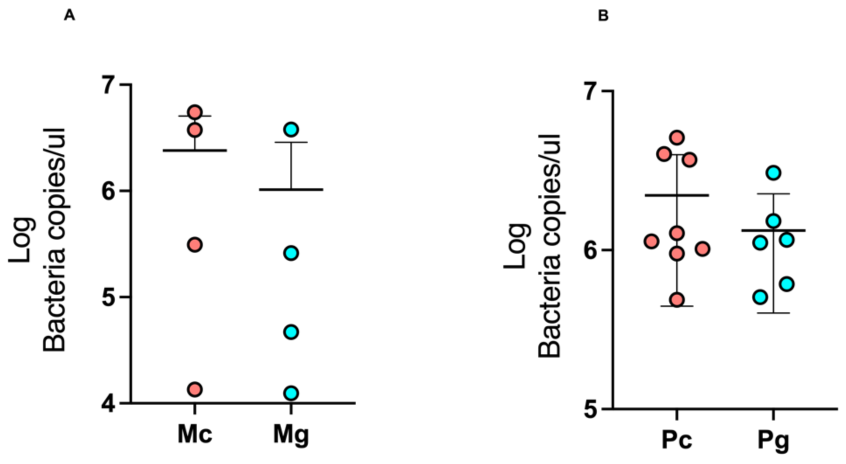

Figure S1:BactQuant analyses of bacteria load in dams and pups. **(A)** Bacteria load in Mc and Mg dams during pregnancy (gestation day 20) **(B)** Bacterial load in Pc and Pg pups at postnatal day 14. Data shown as mean ± SD. **Related to Figure 1.**

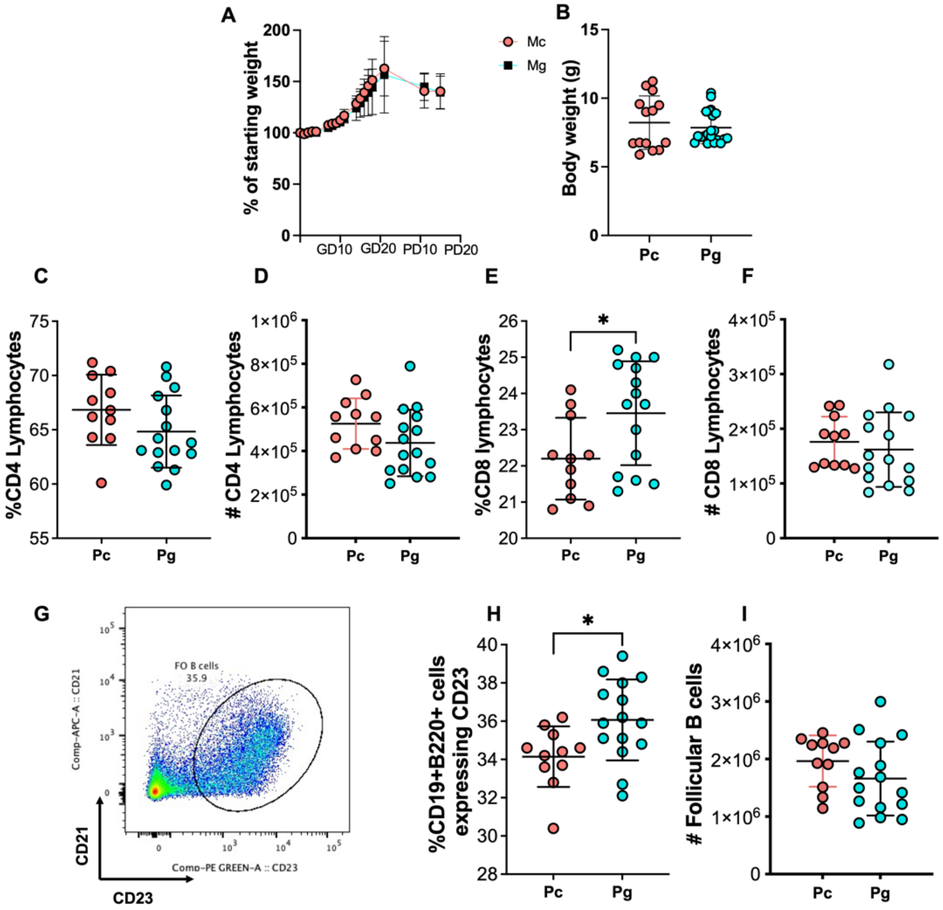

Figure S2: Body weights and Inherent immunity in pups spleens day 14 postpartum. **(A)** Body weight of dams during gestation and at postnatal day 10 and 14. (**B)** Pups body weight at postnatal day 14. (**C-D)** Frequency and numbers of CD4+ T cells. **(E-F)** Frequency and numbers of CD8+ T cells. **(G)** Representative flow plot of Follicular B cells (CD19+B220+CD23+). **(H-I)** Frequency and number of Follicular B cells. Shown as mean ± SD. Data are combined from two independent experiments. n=11-21 per group for pups and 6 per group for dams.*$p < 0.05$. **Related to Figure 2.**

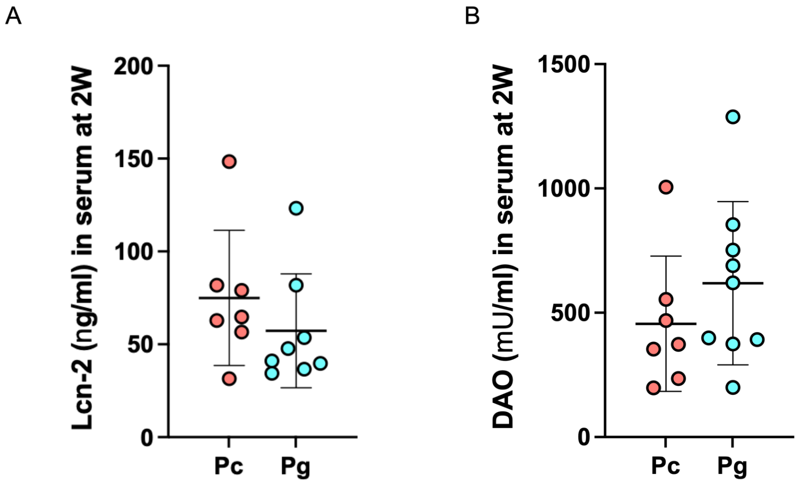

Figure S3: Inflammation and intestinal injury in pups. We measured markers of systemic inflammation or intestinal mucosal injury in pups serum at day 14 of life. (**A)** Concentrations of Lipocalin-2 in serum. (**B)** Concentrations of Diamine Oxidase in serum. Graphs are shown as mean ± SD. N= 7-8 per group. **Related to Figure 2.**
